# Cases Occurring at Union Chapel Hospital, Washington, D. C., 1862

**Published:** 1863-12

**Authors:** W. H. Butler


ART. IV.—*Cases occurring at Union Chapel Hospital, Washington, D. C.*, 1862.—*By* W. H. BUTLER, M. D., *late in charge.*

GUN-SHOT WOUND OF RIGHT FORE-ARM—AMPUTATION—DEATH BY PYÆMIA.—O. L. Otis, aged 25, private Co. D, 83d Pa.; strong and robust, never sick. Was wounded at Gaine's Hill, June 29th, 1862. Wound not dressed till second day, when he started for the James River, traveling 25 miles during the day and night, carrying his shattered arm as well as he could with the other supported by a handkerchief; it bled considerable on the way and he was greatly exhausted on his arrival. Took steamboat for Fortress Monroe, where he was in hospital five days. Admitted to Union Chapel Hospital, Washington, July 7, (a very hot day). Arm laid on a pillow, with simple dressing. Ball had struck the middle of the radius, fracturing it, passing obliquely upwards and out near the inner condyle of the humerus; so as to leave a question whether this process might not be injured, and the joint involved. The wound of entry long and jagged, loose pieces of bone easily felt, the attempt to remove them causing free hemorrhage. Both wounds suppurating freely. Ulna not injured. He had also received a flesh wound, left thumb, and last phalanx 2d and 3d fingers of left hand. No discoloration of arm. Appetite and spirits good.

At a consultation July 9th, present Medical Inspector Coolidge, U. S. A., Surgeon D. W. Bliss, U. S. V., Dr. Stone, of Washington, and house surgeons Butler and Kennon; it was unanimously agreed to amputate the arm.

Patient more irritable than before; says he feels pretty well, but nervous; pulse 120. Dr. Coolidge amputated, making anterior and posterior flaps; middle 3d. Dr. Bliss held the artery; little loss of blood; four arteries tied; found high division of the brachial. Patient under ether. Came out of the operation well. R. Tr. Opii. m. xl. Repeated at bed-time; slept well.

July 10; feels pretty well this morning; not much appetite, but craves drink; stump looks well; ℞ meat broth, tea, &c.; pulse 130; bowels confined; sulph. magnesia ℥i., followed in the evening with enema, sulph. magnesia and water, which relieved the bowels.

Some suppuration was manifest, on the 11th, the pulse had fallen to 100, meantime progressed well; on the 14th the pulse 94; felt well, good appetite; took out all the stitches, supporting it well with straps. On the 19th he sat up; 3 ligatures came away, and on the 29th, the 4th and last.

August 7; has been recovering very rapidly and for about a week has been about the house; stump nearly healed, a small point only uncovered; very little suppuration has occurred for a week past, as much however as the size of the wound would warrant. Now he complains of feeling rather cold, headache, &c.; ℞ S. Quinia. grs. v. Opii. pulv. gr ss., night and morning; says he has been subject to fever and ague. 8th—bowels costive; ℞ mass. hydrarg, gr. x. at 12; evening, seidlitz powder, followed by free dejection.

Had a hard chill later in the evening followed with fever and perspiration, ℞ S. Quinia, grs. v. every 4 hours. 9th; no appetite; pulse 110; feels hot; tongue covered with a white fur; anxious expression of countenance.

Heretofore we had only suspected, now we had the realization that our patient had Pyæmia. ℞ S. Quinia gr. ij. hydrarg chl. mite. gr. i. at 10 o'clock; diet: rice, milk, toast, tea; nausea and want of appetite were present. After this, began to vomit yellowish water; complains of thirst; is in a very irritable, nervous state. 3 P. M, ℞ Bismuth. sub. nit. gr. ij. Morph. gr ss. Morphia to be repeated as he vomits; Turpentine stupe over bowels; S. Quinia in sol. aromat. sulph. acid gr. v. at 4 and 6 P. M. and 2 A.M. 10th; tongue nearly cleared off, not so dry; looks sallow over body; complains of back of head; vomits occasionally, even in taking fluid in small quantity. 9 A. M. Pulse 144; feels hot and thirsty; ℞ Liquor, Ammo. acetas. ℨ ij. Spts. Etheris, Nitrici. gutt. x. every 2 hours; milk, ℥ vj. brandy ℥ j. sweetened to taste with ice every 2 hours. Beef essence ℥ ij. every 4 hours. 7½ P. M.; pulse 140 and feeble; slightly delirious; delirium increased, and he died in active delirium 11½ P. M., from Pyæmia.

The weather for several days has been oppressively hot, the thermometer ranging from 90° to 100° Farenheit.

Here is a case where the patient had apparently passed beyond any chance of untoward result, cut down by this insidious poison.

Amputation of Forearm.—Robert Walker, aged 26, of Co. F, 71st Pa., always healthy and robust, but of rather intemperate habits, received gun-shot wound of left wrist, at battle of White Oaks, June 30th, 1862, the ball entering upper part of wrist, apparently over os magnum, and passing obliquely forwards and downwards, coming out near articulation of metacarpal bone of thumb with wrist; the point of entrance at the time of reception at this hospital, July 7th, being the larger wound.

July 7.—The arm was irrigated with cold water, and it seemed in a fair way to make a good recovery up to the 14th, when he began to complain of pain, and some swelling became apparent; could feel some roughness on introducing the probe.

July 17.—Dr. Coolidge cut down to and removed the os magnum trapezium, unciform, head of third metacarpal bone, head of second metacarpal and sawed off the shaft of second metacarpal for $\frac{3}{4}$ of an inch to get rid of the roughened end. Wound stuffed with lint, and cold water applied. Had bad hiccough in the night; says he is subject to them. ℞. Morph. gr $\frac{3}{4}$, whiskey ℥j.

July 18.—Felt more comfortable; tongue covered with yellowish fur; pulse 98; cold water applied to arm; Sulph. Morph. gr. ss. at night; hand swelling some; looks pale, and edges of cut look dark and unpromising; considerable unpleasant smell; lint not removed; dressed, ung. resinæ, ℞, Sulph. Quinia gr. j, pulv. Opii. gr. ss. every $2\frac{1}{2}$ hours; good diet; Whiskey ℥j, morning and afternoon; slightly delirious Sunday.

July 20.—Hiccough returned some more this morning; ℞ pulv, Opii. grs. v. Ammo Carb. ij ʒ Ft chart No x; 1 every 2 hours, alternating with 1 table spoonful of the following: ℞, Spirits Vini Gallici ʒ vj, Aqua pur ℥iij; beef essence every third interval of brandy; for hiccough ℞, Sulph Etheris comp. ʒj Ammon Aromat gutt x, Morph. gr. $\frac{1}{4}$ every 3 hours. On examining the under part of the arm this morning; found a thick round collection of coagulated blood near the radial artery, above radio carpal articulation, aneurism suspected; the clot was removed, when it began to bleed pretty freely; restrained it by pressing the brachial for a few moments; compression continued for half an hour when it was discontinued, and watched closely. After 2 hours it commenced bleeding again; restrained by compression; pulse 4 P. M. 156; at 5 P. M Dr. Coolidge saw him and on consultation decided to amputate at the upper third of fore-arm. Present, Dr. Charles Bigelow, A. Asst. Surgeon U. S. A, Medical Inspector

Coolidge, Drs. Butler and Kennon, House Surgeons. Patient under ether; circular operation; 4 arteries tied; flaps left open a few moments to guard against hemorrhage as there was some inflammation in the arm up to the point of incision; 6 sutures applied; adhesive plaster and bandage to support stump; patient behaved very well under the effects of the ether; much better than at last operation; after operation ℞ Tr. Opii. m. xl.

July 21.—Patient restless; mutters some; tongue rather thick and coated; pulse 100 and not so full.

Pathological Condition.—Found diffused aneurism coming from radial artery near articulation of wrist, the blood having diffused itself into the muscular tissue about the facia of the wrist and hand. The first row of carpal bones perfect, and trapezoid of second row; there was fracture of third metacarpal bone near its articulation with unciform obliquely upwards and the piece remaining attached; wound looking badly and having a peculiar bad smell.

Treatment; whiskey ℥j every 2 hours, beef tea ℥ iv every 4 hours; at night whiskey ℥ j every 3 hours, to take S. Ether comp. ʒ j Aromat. Sprt. Ammo gutt x Sulph. Morph gr ¼ every 3 hours for hiccough (Typhoid symptoms apparent.)

22d.—Treatment continued to 26th, when Tr. Valerian was substituted for the hiccough and with advantage, a few drachm doses effectually controlling it. The ammonia and morphia only restraining it for a short time.

27th.—Quinia grs. iij, pulv. Ipecac gr. ¼, every 4 hours, whisky ℥j every 3 hours; chicken broth; eats some at each regular meal.

29th.—Treatment continued, oil terebinthe ʒ iss, Sac alba Acacia pulv. q. s, aqua menth ℥i, teaspoonful every 2½ hours. Bed-sores noticed this forenoon over sacrum and on left nates; cataplasm, Lini and Carbon. Eats eggs, chicken, milk, etc., at nearly every meal.

August 2d.—Removed slough from bed.sores, Inject cupri. sulph. gr. ij, wash the back with tannin gr. v, aqua ℥j, Spts. Vini Gallici ʒi, every 2 hours; general treatment continued, but he gradually failed, aud died 12½ o'clock August 10th, The weather for two days previous was very hot—thermometer ranging from 90° to 100° Farenheit.

---

The discussion on hydrophobia at the Academy of Medicine has resulted in the appointment of a rabies committee, charged with the duty of investigating prophylactic measures, and endeavoring to procure the extinction of this scourge of the canine race.

